# Receptor variability-driven evolution of snake toxins

**DOI:** 10.24272/j.issn.2095-8137.2018.063

**Published:** 2018-07-25

**Authors:** Xian-Hong Ji, Shang-Fei Zhang, Bin Gao, Shun-Yi Zhu

**Affiliations:** 1 Group of Peptide Biology and Evolution, State Key Laboratory of Integrated Management of Pest Insects and Rodents, Institute of Zoology, Chinese Academy of Sciences, Beijing 100101, China; 2 University of Chinese Academy of Sciences, Beijing 100049, China

**Keywords:** Three-finger toxins, Nicotinic acetylcholine receptor, Driver

## Abstract

Three-finger toxins (TFTs) are well-recognized non-enzymatic venom proteins found in snakes. However, although TFTs exhibit accelerated evolution, the drivers of this evolution remain poorly understood. The structural complexes between long-chain α-neurotoxins, a subfamily of TFTs, and their nicotinic acetylcholine receptor targets have been determined in previous research, providing an opportunity to address such questions. In the current study, we observed several previously identified positively selected sites (PSSs) and the highly variable C-terminal loop of these toxins at the toxin/receptor interface. Of interest, analysis of the molecular adaptation of the toxin-recognition regions in the corresponding receptors provided no statistical evidence for positive selection. However, these regions accumulated abundant amino acid variations in the receptors from the prey of snakes, suggesting that accelerated substitution of TFTs could be a consequence of adaptation to these variations. To the best of our knowledge, this atypical evolution, initially discovered in scorpions, is reported in snake toxins for the first time and may be applicable for the evolution of toxins from other venomous animals.

## INTRODUCTION

Three-finger toxins (TFTs) are among the most abundantly secreted and effective components of snake venom. They are encoded by a large multigene family and show a diversity of functional activities (Fry et al., 2003; Kini, 2011). Members in this family contain 57–82 residues and four conserved disulfide bridges (Endo & Tamiya, 1987), and are identified by three loops extending from a central core resembling their namesake three fingers (Fry et al., 2003).

The long-term evolutionary process that has resulted in the high diversity of TFTs conforms to the birth-and-death model of multigene family evolution (Nei et al., 1997). This model of evolution produces a series of toxins, allowing snakes to adapt to a variety of prey and predators (Nei et al., 1997). Snakes employ their venom as a weapon to disable prey (primary function) and as a defensive tool against predators (secondary function) (Kang et al., 2011). Snake toxins and prey are likely involved in a co-evolutionary arms race, whereby evolving toxin resistance in prey species and novel toxin evolution in snake species exert mutual selective effects (Arbuckle et al., 2017; Calvete, 2017; Jansa & Voss, 2011; Voss & Jansa, 2012). However, despite evidence of accelerated evolution in the TFT family (Sunagar et al., 2013), the cause of this evolution remains vague.

The TFT family members can target various receptors and ion channels with high affinity and specificity (Doley et al., 2009; Kini, 2011). The α-neurotoxins (α-NTXs) of TFTs can interact with nicotinic acetylcholine receptors (nAChRs), inhibiting postsynaptic membrane ion flow and leading to flaccid paralysis (Barber et al., 2013; Chiappinelli et al., 1996). Based on the chain length and number of disulphide bridges, α-NTXs are usually divided into short or long α-NTXs. Short α-NTXs contain 60–62 amino acid residues with four disulfide bonds, whereas long α-NTXs contain 66–75 residues and five disulfide bonds (Dajas-Bailador et al., 1998).

nAChRs are pentameric transmembrane proteins of ligand-gated ion channels and are formed by different combinations of five subunits, that is, α, β, γ, δ and ε (Corringer et al., 2000; Karlin, 2002). Each subunit is composed of a large N-terminal extracellular domain, which serves as the major binding site for the toxins, followed by four transmembrane helices and a small C-terminal extracellular domain (Dellisanti et al., 2007; Karlin, 2002). nAChRs can be further divided into muscular or neuronal types (Corringer et al., 2000; Wang et al., 2002). α-NTXs can potently antagonize the α1 subunit of heteropentameric muscle nAChRs ((α1)_2_β1γδ in fetal muscle and (α1)_2_β1εδ in adult muscle) and the α7, α8, α9 or α10 subunits of homopentameric neuronal nAChRs (Karlin, 2002; Sine et al., 2013). Crystal structures in the complexes between long α-NTXs and related receptors have been identified and offer opportunities to explore the molecular mechanism driving the accelerated evolution of these toxins (Bourne et al., 2005; Dellisanti et al., 2007; Huang et al., 2013).

In this work, we found several sites previously identified as positively selected sites (PSSs) as well as the highly variable C-terminal loop of the toxins to be located at the toxin/receptor binding interface (Bourne et al., 2005; Sunagar et al., 2013). Structural and evolutionary analyses of the toxin-recognition region from the receptors (α1, α7, α9 and α10 subunits from nAChRs) uncovered an atypical evolution between snakes and their prey, in which the amino acid diversity of the nAChR toxin-binding regions appeared to drive the adaptive evolution of the TFT family. This study showed good agreement with our previous research on scorpion toxins and sodium channels from their competitors (Zhang et al., 2015). Furthermore, this paper provides a broader vision into the evolution between venomous animals and their prey/predators.

## MATERIALS AND METHODS

### Sequence analysis

ClustalX (http://www.clustal.org) was used to align all nucleotide and amino acid sequences. A total of 130 long α-NTX sequences were aligned and used for sequence logo analysis with the WebLogo program (Supplementary Figure S1). For the receptors, 76 sequences of muscle-type α1 nAChRs (three from *Reptilia*, three from *Amphibian*, four from *Aves*, 31 from small mammals, and 35 from fishes), 59 neuronal-type α7 nAChRs (one from *Gastropoda*, two from *Arthropoda*, three from *Reptilia*, three from *Amphibian*, three from *Aves*, 18 from small mammals, and 29 from fishes), 68 neuronal-type α9 nAChRs (three from *Reptilia*, three from *Amphibian*, four from *Aves*, 30 from small mammals, and 28 from fishes), and 52 neuronal-type α10 nAChRs (two from *Reptilia*, two from *Amphibian*, four from *Aves*, 24 from small mammals, and 20 from fishes) (Supplementary Figures S2–S5) were also aligned. For positive selection analyses, aligned nucleotide sequences of the receptors were used to construct neighbor-joining trees.

### WebLogo and ConSurf analyses

WebLogo can be used to generate sequence logos, which are graphical representations of the patterns within multiple sequence alignments. The alignments of the aforementioned toxins were used to create sequence logos to identify the conservation of each position (Supplementary Figure S1). Each logo comprises stacks of letters, one stack for each position in the sequence (Crooks et al., 2004). The overall height of each stack shows the sequence conservation of that position, whereas the height of the symbols within the stack indicates the comparative frequency of the amino acid at the position (Crooks et al., 2004). Generally, sequence logos provide a richer and more precise description of conserved and variable regions within sequences. We used the ConSurf program (http://consurf.tau.ac.il/) under default parameters to calculate conservation scores of the amino acid sequences of the related receptors. ConSurf not only depends on sequence alignments but also on phylogenetic trees to identify conserved and variable regions (Landau et al., 2005). A Bayesian tree was constructed using the corresponding alignments with the JTT evolutionary substitution model. One of the advantages of ConSurf compared with other methods is that the computation of the evolutionary rate is more precise when employing the empirical Bayesian or maximum-likelihood methods.

### Positive selection analysis

Excess nonsynonymous substitutions compared with synonymous substitutions (ω=*d*_N_/*d*_S_>1) is an important sign of positive selection at the molecular level (Yang, 1998; Zhu & Gao, 2016). To perform such analysis, we compared two pairs of site models (M1a (neutral)/M2a (selection) and M7 (beta)/M8 (beta & ω)) to measure the selective pressure of the receptors to which the long α-NTXs bind (α1, α7, α9 and α10 subunits from nAChRs). Model M2a and M8 add a site class to M1a and M7, respectively, with the free ω ratio calculated from the data and used to determine the probability of positive selection (Anisimova et al., 2001; Anisimova et al., 2003; Wong et al., 2004; Yang & Swanson, 2002). As M1a and M7 are nested within their respective alternative models (M2a and M8) and have two more parameters, χ^2^ distribution can be used for the likelihood ratio test to compare the fit of the two competing models. We used the Bayes Empirical Bayes method to calculate the posterior possibility that each codon is from the site class of positive selection. The Bayes Empirical Bayes method is an improvement of the previous Naïve Empirical Bayes method and accounts for sampling errors in the maximum-likelihood estimates of parameters in the model (Nielsen & Yang, 1998). Sites with a high possibility (≥95%) of coming from the class with ω>1 are likely under positive selection and can be analyzed further (Yang, 1998).

## RESULTS

### PSSs of toxins locating on the toxin-receptor complex interface

**Figure 1:**
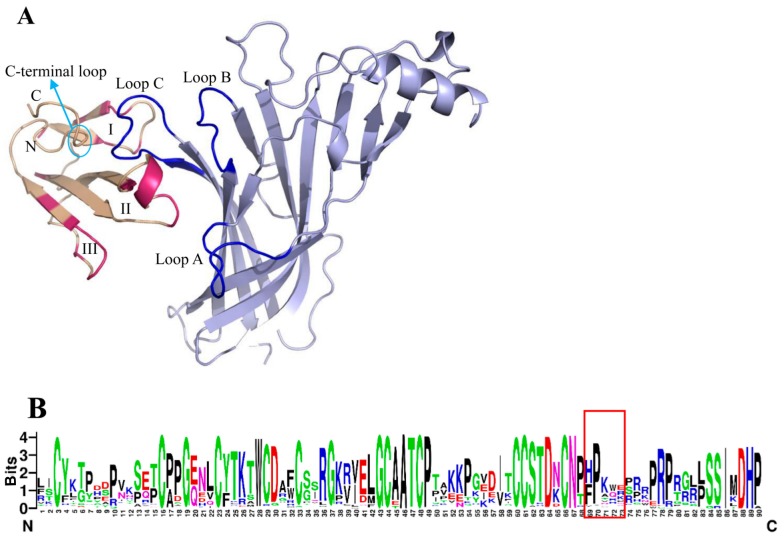
α-bungarotoxin interacts with α211 and the sequence logos of long α-NTXs

The N-terminal extracellular domains of nAChRs, which consist of a 10 stranded β-sandwich and an N-terminal α-helix, act as the major binding sites for long α-NTXs (Changeux et al., 1970). The three regions of long α-NTXs comprising fingers I and II and the C-terminus are involved in interactions with the receptors, with finger II being the main stabilizing interaction center (Bourne et al., 2005). The α211 structure (mouse nAChR α1 subunit (PDB entry 2qc1)) can be seen as a representative of the N-terminal extracellular domain of the nAChR subunit (Dellisanti et al., 2007), in which loops β4-β5 (loop A), β7-β8 (loop B) and β9-β10 (loop C) serve as the principal ligand-binding interfaces (Brejc et al., 2001; Unwin, 2005) and loop C is the most important region for high affinity with long α-NTXs, as revealed by site-directed mutations (Fruchart-Gaillard et al., 2002; Levandoski et al., 1999). Loop C of α211 is enveloped by fingers I and II and the C-terminal loop of the toxin, with finger II inserted into the ligand-binding site wrapped by loops A, B and C of α211 (Figure 1A) (Dellisanti et al., 2007). In addition, finger I is sandwiched by loop C, whereas finger III weakly contributes to α211 binding (Dellisanti et al., 2007).

A: Interaction between α-bungarotoxin (long α-NTXs) and α211. α-bungarotoxin is shown in wheat and α211 in light blue. The PSSs of the long α-NTXs are in pink. The three loops of α211, major toxin-binding regions, are highlighted in blue. B: Sequence logos of long α-NTXs. The C-terminal loop of the long α-NTXs involved in receptor binding is indicated in a red box.

We mapped the PSSs of long α-NTXs identified by Sunagar et al. (2013) on the complex structure of α-bungarotoxin and its receptor (Figure 1A). The toxin-receptor complex model showed that some PSSs of the long α-NTXs were located on the toxin binding surface with the receptors (Bourne et al., 2005; Dellisanti et al., 2007; Huang et al., 2013). Almost all PSSs in finger II (Ala31, Phe32, Ser34, Ser35 and Val39) of α-bungarotoxin are involved in the interactions with the receptors (Dellisanti et al., 2007; Dimitropoulos et al., 2011; Huang et al., 2013). The same situation appears in α-cobratoxin (long α-NTX) since their fast evolved sites (Ala28, Phe29, Ser31, Ile32 and Arg36) in finger II also overlap with the toxin binding sites of α-cobratoxins (Bourne et al., 2005; Dimitropoulos et al., 2011).

The C-terminal loop of the long α-NTXs also contributes to the interaction with related receptors. Multiple sequence alignments of long α-NTXs, generated by ClustalX, were used to create sequence logos (Figure 1B, Supplementary Figure S1) (Crooks et al., 2004). The C-terminal loop of the long α-NTXs, indicated in the red box in Figure 1B, was highly variable based on the WebLogo analyses, and the whole C-tail involves extensive insertions and deletions. Thus, this loop might undergo positive selection despite the technical difficulty in detecting PSSs from indel-containing sequences.

### No evidence for positive selection in the toxin-recognition regions of receptors

Snakes employ their venom to immobilize various prey, including snails, insects, fishes, toads, lizards, chickens, small mammals and even other snakes (Kang et al., 2011). The maximum-likelihood models of codon substitutions were used to identify selective pressure in the toxin-recognition regions of the receptors from the prey of snakes. However, no positive selection signals were detected in the receptors of long α-NTXs (α1, α7, α9 and α10 subunits) (Table 1, Supplementary Tables S1–S4). The maximum-likelihood estimates under M0 showed that the average ω ratios for all receptor sequence pairs ranged from 0.02 to 0.07. M2a and M8 detected no evolution-accelerating sites. The ω_s_ under M8 of the α1 and α10 subunits of nAChRs from snake prey equaled 1 (Table 1). Under the M8 model, the α7 and α9 subunits of nAChRs showed ω_s_>1 (Table 1), but their proportions (p1) equaled 0, proving that no PSSs existed (Supplementary Tables S1–S4).

**Table 1:** Parameter estimates and likelihood ratio statistics (2Δ*l*) for different subunit types of nAChRs

Type	*l* (M1a)	*l* (M2a)	$\omega_{2}$ (M2a)	2Δ*l*	*l* (M7)	*l* (M8)	ω_s_ (M8)	2Δ*l*
α1 subunit	–12874.0	–12874.0	**1.00**	0	–12617.4	–12607.4	**1.00**	20
α7 subunit	–13163.9	–13163.9	**1.00**	0	–12785.7	–12785.7	**34.66**	0
α9 subunit	–11856.3	–11856.3	**1.00**	0	–11626.9	–11626.9	**1.42**	0
α10 subunit	–9954.2	–9954.2	**1.00**	0	–9746.8	–9746.8	**1.00**	0

*l* is the log likelihood; 2Δ*l* is between null models and their alternative models: M1a/M2a and M7/M8. Two ω values in M2a and M8 are boldfaced.

### Snake toxins bind to variable regions of their receptors

**Figure 2:**
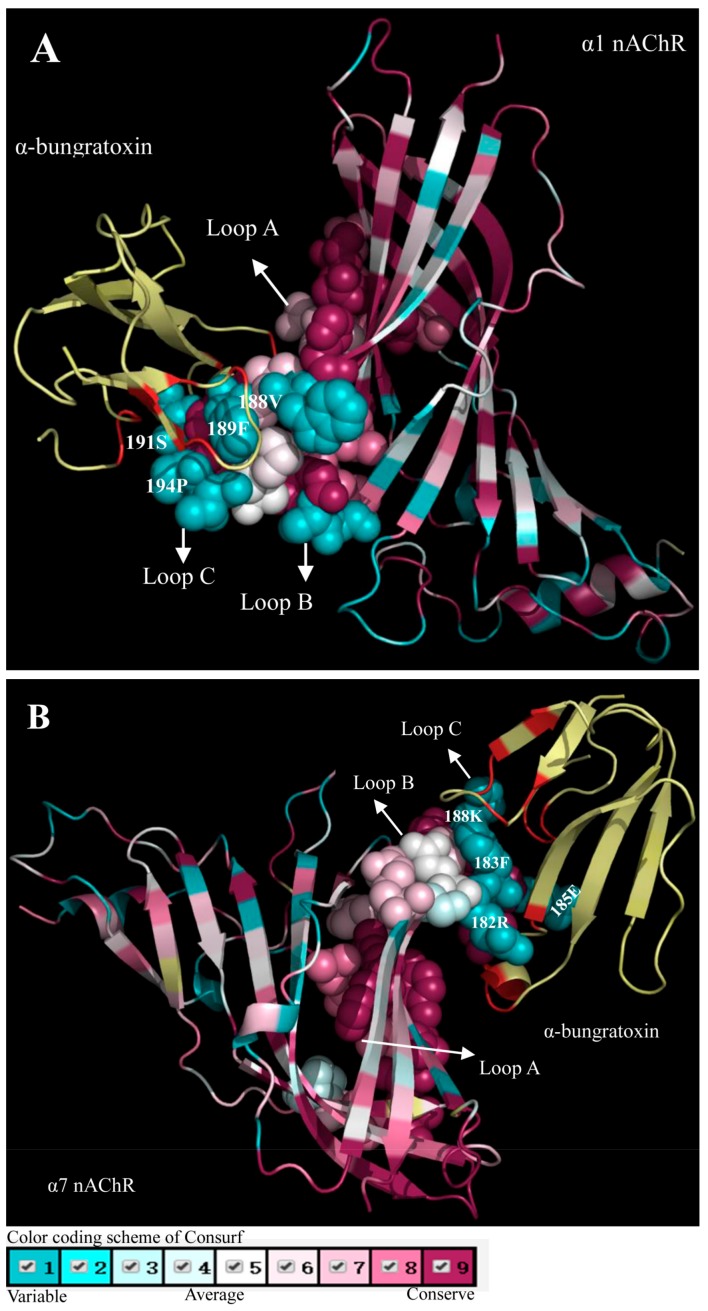
ConSurf results of α-bungarotoxin with the receptors

Although PSSs were detected in the toxins, none were found in the toxin-recognition regions of the involved receptors. Based on the complex models between the long α-NTXs and related receptors, we further analyzed the evolutionary conservation of the N-terminal extracellular domain regions of the nAChR α1 and α7 subunits from snake prey using ConSurf (Dellisanti et al., 2007; Huang et al., 2013) (Figure 2). Our results indicated that loop C demonstrated the greatest variation among the three receptor loops (Figure 2).

ConSurf results of α-bungarotoxin with α1 (PDB entry 2qc1) and α7 (PDB entry 4hqp) nAChRs are shown in A and B, respectively. ConSurf provides evolutionary conservation scores for nAChRs. PSSs of finger I and finger II and the residues of the C-terminal loop in α-bungarotoxin are highlighted in red (PSSs of finger II are partly hidden). Toxin-binding regions (loop A, loop B and loop C) of the α1 and α7 nAChRs are spherized. Binding sites variable in loop C are indicated.

We analyzed the interaction pairs in the α-bungarotoxin and α1 nAChR subunit complex, which were Ile11-Phe189/ Pro194, Val31-Tyr93/Asp99/Phe100, Phe32-Tyr93/Phe100/ Trp149/Tyr190, Val39-Val188/Tyr190, His68-Pro194, Pro69- Ser191, Lys70-Pro194 and Gln71-Pro194, with the toxin sites corresponding to the PSSs in fingers I and II and the C-terminal loop (Dellisanti et al., 2007; Dimitropoulos et al., 2011). The loop A (Tyr93, Asp99 and Phe100), loop B (Trp149) and loop C (Val188, Phe189, Tyr190, Ser191, Pro194 and Pro197) were involved in the interaction with the PSSs of the toxin binding surface. Although Tyr93 in loop A and Trp149 in loop B were conserved, Asp99 and Phe100 in loop A exhibited some variability (99: Asp/Asn and 100: Phe/Tyr). In contrast, the binding sites in loop C were more variable (188: Val/Arg/Lys, 189: Phe/Tyr/Thr, 191: Ala/Ser/Thr/Pro and 194: Pro/Gln) (Figure 2A).

For the α-bungarotoxin and α7 nAChR subunit complex, the interaction pairs include Ile11-Phe183/Lys188, Phe32- Tyr91/Trp145/Arg182/Tyr184, Val39-Arg182/Tyr184, His68- Lys188, Pro69-Glu185, Lys70-Cys186/Lys188 and Gln71- Lys188, with the sites of the toxins also corresponding to the PSSs in fingers I and II and the C-terminal loop (Huang et al., 2013). Compared with the conserved residues in loop A and loop B (Tyr91 and Trp145), the binding sites in loop C were diverse (182: Lys/Arg/Leu/Ser/Asn, 183: Phe/Tyr/Ile, 185: Glu/Asp/Asn/Gly and 188: Lys/Asp/Glu/Pro/Gln) (Figure 2B). The additional PSSs (Val31, Ser34 and Ser35) in finger II contacted residues of the complementary subunit, and included Ser32, Ser34, Leu36, Trp53, Gln55, Gln114, Leu116 and Asp160 (Huang et al., 2013). These residues in the complementary subunit may provide the driving force for the additional sites in finger II.

Taken together, our results suggest that the variable toxin-recognition region in the receptors might drive the accelerated evolution of the toxin-binding residues.

## DISCUSSION

By mapping the PSSs of the long α-NTXs on the toxin-receptor complex, we found that several of them are located on the toxin-binding surface of the receptor (Figure 1A). In addition to these PSSs, high sequence diversity was also observed in the C-terminal loop of the long α-NTXs. Thus, given its importance in the interaction with receptors, we surmised that it could be an accelerated substituted region for adaptation to receptor variability (Figure 1B). Several PSSs were detected in finger III of the long α-NTXs, although finger III contributed little to the binding. This may be due to its high flexibility in the complex. Other mechanisms may be involved in the accelerated substitutions of this finger, which requires further investigation.

We further observed the amino acid variability of the principal toxin-recognition regions (mainly in loop C) in related nAChR subunits (Figure 2). Compared with loop A and loop B, loop C exhibited the greatest variation due to its predominant role in the toxin-receptor interactions. Thus, we concluded that the evolutionary variability of the toxin-recognition regions of the receptors is a possible driver for the accelerated evolution of the toxins.

Short-chain α-NTXs can bind to nAChRs with high affinity (Trémeau et al., 1995). Loop II of short-chain α-NTXs pushes into the ligand-binding pocket of nAChRs, whereas the tips of loops I and III contact nAChRs only in a ‘surface-touch’ way (Mordvintsev et al., 2005). Previous study on *Torpedo californica* showed that the C-loop is vital for the binding of short α-NTXs to nAChRs (Bourne et al., 2005; Mordvintsev et al., 2005). However, as there are no specific coordinate files of complexes between short α-NTXs and their targets, hindering further study.

Our previous study on the evolution of scorpion toxins and voltage-gated sodium (Na_v_) channels indicated that variability of the toxin-recognition regions in the Na_v_ channels from scorpion predators and prey is a putative driver of the accelerated evolution of the functional regions of scorpion toxins following gene duplication (Zhang et al., 2015). Similarly, PSSs exist in the binding interface of the long α-NTXs, though only amino acid variability was detected in the principal toxin-recognition regions of the related nAChR subunits, suggesting that amino-acid substitutions on the toxin-recognition surface in related nAChR subunits could provide a force driving the accelerated evolution of the toxins. Thus, the atypical co-evolutionary manner between snake toxins and their receptors is similar to our previous research on scorpion toxins and their targets (Zhang et al., 2015). We supposed that accelerated evolution of the receptor-bound regions of the snake toxins is a consequence of adaptation to variable receptors of their prey. From the viewpoint of a co-evolutionary arms race between predators and prey (Arbuckle et al., 2017; Calvete, 2017; Jansa & Voss, 2011; Voss & Jansa, 2012), it appears that prey might exert greater selective pressure on their predators, as described in our current study. As this evolutionary manner has been shown to occur in two distant species, we believe it will be revealed in more toxins from diverse venomous animals.

## Supplementary Material

http://www.zoores.ac.cn/fileup/2095-8137/SUPPL/20180803181809.zip

## References

[B1-ZoolRes-39-6-431] Anisimova M., Bielawski J.P., Yang Z.H. (2001). Accuracy and power of the likelihood ratio test in detecting adaptive molecular evolution. Molecular Biology and Evolution.

[B2-ZoolRes-39-6-431] Anisimova M., Nielsen R., Yang Z.H. (2003). Effect of recombination on the accuracy of the likelihood method for detecting positive selection at amino acid sites. Genetics.

[B3-ZoolRes-39-6-431] Arbuckle K., Rodriguez De La Vega R.C., Casewell N.R. (2017). Coevolution takes the sting out of it: Evolutionary biology and mechanisms of toxin resistance in animals. Toxicon.

[B4-ZoolRes-39-6-431] Barber C.M., Isbister G.K., Hodgson W.C. (2013). Alpha neurotoxins. Toxicon.

[B5-ZoolRes-39-6-431] Bourne Y., Talley T.T., Hansen S.B., Taylor P., Marchot P. (2005). Crystal structure of a Cbtx-AChBP complex reveals essential interactions between snake alpha-neurotoxins and nicotinic receptors. The EMBO Journal.

[B6-ZoolRes-39-6-431] Brejc K., Van Dijk W.J., Klaassen R.V., Schuurmans M., Van Der Oost J., Smit A.B., Sixma T.K. (2001). Crystal structure of an ACh-binding protein reveals the ligand-binding domain of nicotinic receptors. Nature.

[B7-ZoolRes-39-6-431] Calvete J.J. (2017). Venomics: Integrative venom proteomics and beyond. The Biochemical Journal.

[B8-ZoolRes-39-6-431] Changeux J.P., Kasai M., Lee C.Y. (1970). Use of a snake venom toxin to characterize the cholinergic receptor protein. Proceedings of the National Academy of Sciences of the United States of America.

[B9-ZoolRes-39-6-431] Chiappinelli V.A., Weaver W.R., Mclane K.E., Conti-Fine B.M., Fiordalisi J.J., Grant G.A. (1996). Binding of native κ-neurotoxins and site-directed mutants to nicotinic acetylcholine receptors. Toxicon.

[B10-ZoolRes-39-6-431] Corringer P.J., Le Novère N., Changeux J.P. (2000). Nicotinic receptors at the amino acid level. Annual Review of Pharmacology and Toxicology.

[B11-ZoolRes-39-6-431] Crooks G.E., Hon G., Chandonia J.M., Brenner S.E. (2004). WebLogo: A sequence logo generator. Genome Research.

[B12-ZoolRes-39-6-431] Dajas-Bailador F., Costa G., Dajas F., Emmett S. (1998). Effects of alpha-erabutoxin, alpha-bungarotoxin, alpha-cobratoxin and fasciculin on the nicotine-evoked release of dopamine in the rat striatum in vivo. Neurochemistry International.

[B13-ZoolRes-39-6-431] Dellisanti C.D., Yao Y., Stroud J.C., Wang Z.Z., Chen L. (2007). Crystal structure of the extracellular domain of nAChR alpha1 bound to alpha-bungarotoxin at 1.94 A resolution. Nature Neuroscience.

[B14-ZoolRes-39-6-431] Dimitropoulos N., Papakyriakou A., Dalkas G.A., Chasapis C.T., Poulas K., Spyroulias G.A. (2011). A computational investigation on the role of glycosylation in the binding of alpha1 nicotinic acetylcholine receptor with two alpha-neurotoxins. Proteins.

[B15-ZoolRes-39-6-431] Doley R., Mackessy S.P., Kini R.M. (2009). Role of accelerated segment switch in exons to alter targeting (ASSET) in the molecular evolution of snake venom proteins. BMC Evolutionary Biology.

[B16-ZoolRes-39-6-431] Endo T., Tamiya N. (1987). Current view on the structure-function relationship of postsynaptic neurotoxins from snake venoms. Pharmacology & Therapeutics.

[B17-ZoolRes-39-6-431] Fruchart-Gaillard C., Gilquin B., Antil-Delbeke S., Le Novere N., Tamiya T., Corringer P.J., Changeux J.P., Menez A., Servent D. (2002). Experimentally based model of a complex between a snake toxin and the alpha 7 nicotinic receptor. Proceedings of the National Academy of Sciences of the United States of America.

[B18-ZoolRes-39-6-431] Fry B.G., Wuster W., Kini R.M., Brusic V., Khan A., Venkataraman D., Rooney A.P. (2003). Molecular evolution and phylogeny of elapid snake venom three-finger toxins. Journal of Molecular Evolution.

[B19-ZoolRes-39-6-431] Huang S., Li S.X., Bren N., Cheng K., Gomoto R., Chen L., Sine S.M. (2013). Complex between α-bungarotoxin and an α7 nicotinic receptor ligand-binding domain chimaera. The Biochemical Journal.

[B20-ZoolRes-39-6-431] Jansa S.A., Voss R.S. (2011). Adaptive evolution of the venom-targeted vWF protein in opossums that eat pitvipers. PloS One.

[B21-ZoolRes-39-6-431] Kang T.S., Georgieva D., Genov N., Murakami M.T., Sinha M., Kumar R.P., Kaur P., Kumar S., Dey S., Sharma S., Vrielink A., Betzel C., Takeda S., Arni R.K., Singh T.P., Kini R.M. (2011). Enzymatic toxins from snake venom: structural characterization and mechanism of catalysis. The FEBS Journal.

[B22-ZoolRes-39-6-431] Karlin A. (2002). Emerging structure of the nicotinic acetylcholine receptors. Nature Reviews. Neuroscience.

[B23-ZoolRes-39-6-431] Kini R.M. (2011). Evolution of three-finger toxins - a versatile mini protein scaffold. Acta Chimica Slovenica.

[B24-ZoolRes-39-6-431] Landau M., Mayrose I., Rosenberg Y., Glaser F., Martz E., Pupko T., Ben-Tal N. (2005). ConSurf 2005: The projection of evolutionary conservation scores of residues on protein structures. Nucleic Acids Research.

[B25-ZoolRes-39-6-431] Levandoski M.M., Lin Y., Moise L., Mclaughlin J.T., Cooper E., Hawrot E. (1999). Chimeric analysis of a neuronal nicotinic acetylcholine receptor reveals amino acids conferring sensitivity to alpha-bungarotoxin. The Journal of Biological Chemistry.

[B26-ZoolRes-39-6-431] Mordvintsev D.Y., Polyak Y.L., Levtsova O.V., Tourleigh Y.V., Kasheverov I.E., Shaitan K.V., Utkin Y.N., Tsetlin V.I. (2005). A model for short alpha-neurotoxin bound to nicotinic acetylcholine receptor from Torpedo californica: comparison with long-chain alpha-neurotoxins and alpha-conotoxins. Computational Biology and Chemistry.

[B27-ZoolRes-39-6-431] Nei M., Gu X., Sitnikova T. (1997). Evolution by the birth-and-death process in multigene families of the vertebrate immune system. Proceedings of the National Academy of Sciences of the United States of America.

[B28-ZoolRes-39-6-431] Nielsen R., Yang Z. (1998). Likelihood models for detecting positively selected amino acid sites and applications to the HIV-1 envelope gene. Genetics.

[B29-ZoolRes-39-6-431] Sine S.M., Huang S., Li S.X., daCosta C.J.B., Chen L. (2013). Inter-residue coupling contributes to high-affinity subtype-selective binding of alpha-bungarotoxin to nicotinic receptors. The Biochemical Journal.

[B30-ZoolRes-39-6-431] Sunagar K., Jackson T.N.W., Undheim E.A.B., Ali S.A., Antunes A., Fry B.G. (2013). Three-fingered RAVERs: Rapid Accumulation of Variations in Exposed Residues of snake venom toxins. Toxins.

[B31-ZoolRes-39-6-431] Trémeau O., Lemaire C., Drevet P., Pinkasfeld S., Ducancel F., Boulain J.C., Ménez A. (1995). Genetic Engineering of Snake Toxins. Journal of Biological Chemistry.

[B32-ZoolRes-39-6-431] Unwin N. (2005). Refined structure of the nicotinic acetylcholine receptor at 4A resolution. Journal of Molecular Biology.

[B33-ZoolRes-39-6-431] Voss R.S., Jansa S.A. (2012). Snake-venom resistance as a mammalian trophic adaptation: lessons from didelphid marsupials. Biological Reviews of the Cambridge Philosophical Society.

[B34-ZoolRes-39-6-431] Wang N., Orr-Urtreger A., Korczyn A.D. (2002). The role of neuronal nicotinic acetylcholine receptor subunits in autonomic ganglia: lessons from knockout mice. Progress in Neurobiology.

[B35-ZoolRes-39-6-431] Wong W.S., Yang Z., Goldman N., Nielsen R. (2004). Accuracy and power of statistical methods for detecting adaptive evolution in protein coding sequences and for identifying positively selected sites. Genetics.

[B36-ZoolRes-39-6-431] Yang Z. (1998). Likelihood ratio tests for detecting positive selection and application to primate lysozyme evolution. Molecular Biology and Evolution.

[B37-ZoolRes-39-6-431] Yang Z., Swanson W.J. (2002). Codon-substitution models to detect adaptive evolution that account for heterogeneous selective pressures among site classes. Molecular Biology and Evolution.

[B38-ZoolRes-39-6-431] Zhang S., Gao B., Zhu S. (2015). Target-Driven Evolution of Scorpion Toxins. Scientific Reports.

[B39-ZoolRes-39-6-431] Zhu S., Gao B. (2016). Positive selection in cathelicidin host defense peptides: adaptation to exogenous pathogens or endogenous receptors?. Heredity.

